# Complete Genome Sequence of Paradevosia shaoguanensis Type Strain J5-3, Obtained Using Nanopore and Illumina Sequencing Technologies

**DOI:** 10.1128/MRA.00099-21

**Published:** 2021-06-10

**Authors:** Yang Wang, Songxue Wang

**Affiliations:** a Academy of National Food and Strategic Reserves Administration, Beijing, People’s Republic of China; University of Southern California

## Abstract

The complete genome sequence of Paradevosia shaoguanensis J5-3^T^ (China General Microbiological Culture Collection Center [CGMCC] 1.12430^T^) is presented here. The complete genome sequence of *P*. *shaoguanensis* J5-3^T^ will provide valuable references for classification and comparative genome analysis.

## ANNOUNCEMENT

The genus *Paradevosia*, which was first found in 2015, belongs to the family *Hyphomicrobiaceae* and is a close relative of the genera *Devosia*, *Youhaiella*, and *Pelagibacterium* (1–4). Paradevosia shaoguanensis J5-3^T^ is the type species of the genus *Paradevosia*. However, there has been no genome sequence reported for this microorganism so far. In the present study, we determined the complete genome sequence of Paradevosia shaoguanensis J5-3^T^ to gain a greater understanding of this strain.

*P. shaoguanensis* strain J5-3^T^ (i.e., CGMCC 1.12430^T^ or LMG 27409^T^) was purchased from the China General Microbiological Culture Collection Center (CGMCC). The strain was grown aerobically at 30°C in LB for 24 h, and then its total genomic DNA was extracted using the Wizard genomic DNA purification kit (Promega) following the manufacturer’s instructions. The whole genome was sequenced at Biomaker Technology, Inc. (Beijing, People’s Republic of China) with a combination of the Nanopore PromethION 48 system and the Illumina NovaSeq 6000 platform. For long-read sequencing, library preparation used the ligation sequencing kit 1D (SQK-LSK109) and sequencing used R9.4.1 ONT flow cells on the PromethION 48 device (Oxford Nanopore Technologies [ONT], Oxford, UK). Raw sequence data (fast5 format) were base-called using the Albacore sequencing pipeline version 2.0.2 software, and a total of 130,089 raw reads (*N*_50_, 24,483 bp) were generated. Short (<2,000 bp) and/or low-quality (quality scores of <6) reads were filtered using Filtlong software (https://github.com/rrwick/Filtlong). After elimination of adaptor sequences and low-quality reads, the remaining reads (103,940 reads, with an average read length of 16,423 bp and *N*_50_ value of 24,805 bp) were *de novo* assembled into an initial assembly (4,629,545 bp) with Canu version 1.8 (5). The assembly was circularized using the Minimus2 circularization pipeline (https://github.com/sanger-pathogens/circlator/wiki/Minimus2-circularization-pipeline), and the overlapping sequences were trimmed at the assembly ends (6). For short-read sequencing, a library with an insert size of 350 bp was constructed using a TrueLib DNA library rapid prep kit and sequenced using an Illumina NovaSeq 6000 system. The quality of Illumina reads was assessed using FastQC version 0.11.5, and then these reads (7,881,476 reads) were mapped to the assembly with the Burrows-Wheeler Aligner version 0.7.17 (7) for sequence and assembly error correction with Pilon version 1.2.3 (8). The assembly quality and completeness were assessed using BUSCO version 5.1.2 with the alphaproteobacteria_odb10 database, which showed that the genome sequence is >99.4% complete (9). Default settings were used for all software unless otherwise noted. The genome sequence of *P*. *shaoguanensi*s J5-3^T^ was assembled into a single circular chromosomal contig of 4,629,545 bp (368.74× coverage) with a mean GC content of 63.91%.

The assembled sequence was annotated using the NCBI Prokaryotic Genome Annotation Pipeline (PGAP) (10, 11). The annotation revealed 4,420 protein-coding genes from 4,450 coding DNA sequences (CDSs) in 4,505 genes, with 2 rRNA operons comprising 5S, 16S, and 23S rRNAs, 50 tRNAs, 4 noncoding RNAs, and 25 pseudogenes.

### Data availability.

The full genomic sequence of *P. shaoguanensis* J5-3^T^ has been deposited in NCBI/GenBank under BioProject number PRJNA694805 with GenBank accession number CP068983, BioSample number SAMN17574782, and SRA numbers SRR13528957 (PromethION) and SRR13528956 (Illumina).
